# 

**Published:** 1894-09

**Authors:** 


### CONTENTS:

ORIGINAL ARTICLES: PAGE

The Anatomy of the Large American Fluke (*Fasciola Magna*). By Charles Wardell Stiles, Ph.D. .... 225
Carbuncle of the Coronary Ligament and Neighboring Tissues. By H. McWhinnie, D.V.S .... 244
Purpura Hemorrhagica, Œdema, Anasarca, Etc. By Dr. H. A. Spencer .... 245
Parturient Apoplexy. By F. E. Pierce, D.V.S .... 248

SELECTIONS:

Constitution and By-laws of the Veterinary Medical Association of New York County 250
Graduates, 1894 .... 254

EDITORIAL:

The United States Veterinary Medical Association .... 267
Veterinary Legislation .... 268
Veterinary Education .... 269
Change of Management .... 269

SOCIETY PROCEEDINGS:

German Veterinary Medical Association from New York and Vicinity .... 271
California State Veterinary Medical Association .... 272
Massachusetts Veterinary Association .... 276
Erie County Medical Veterinary Society .... 279
Pennsylvania State Medical Association .... 280
United States Veterinary Medical Association .... 293
An Act to Establish a board of Veterinary Medical Examiners in the State of Pennsylvania .... 294

Obituary .... 292

